# Effect of immunized egg proteins on the performance and neonatal diarrhoea incidence in newborn calves

**DOI:** 10.1111/jpn.13484

**Published:** 2021-01-19

**Authors:** Sandra van Kuijk, Ruth Kinkead, Gillian Scoley, Steven Morrison, Yanming Han

**Affiliations:** ^1^ Trouw Nutrition R&D Amersfoort the Netherlands; ^2^ Sustainable Agri‐Food Sciences Division Agri‐Food and Biosciences Institute (AFBI) Hillsborough UK

**Keywords:** diarrhoea, growth performance, immunized egg protein, newborn calves

## Abstract

The aim of this study was to assess the effects of feeding immunized egg proteins (IEP) on the health and performance of newborn dairy calves. Sixty‐four Holstein calves, both male and female, were divided over two treatments. Calves either received IEP or a placebo (PCB) in their colostrum and calf milk replacer (CMR) for the first 14 days of their life. Until day 49, CMR was offered at 15% of birth weight (BW), at 10% on days 49–57 and at 5% on days 57–63. In addition, calves received starter concentrate, chopped straw and water from 3 days old until 70 days old at the end of study. Individual CMR and concentrate intake were measured daily whilst BW was recorded weekly. Visual faecal scoring and health observations were conducted daily. Faecal samples were collected weekly up to 4 weeks and during the first 4 days of scouring to screen for presence of *Cryptosporidium parvum*, rotavirus, coronavirus, *E. coli* and *Salmonella*. Results indicated that feeding IEP increased BW (*p* < .05) at 42 and 56 days old, and BW also tended (*p* = .06) to be higher after weaning at 63–70 days old compared to the PCB group. When analysed using a repeated measures model, compared to feeding PCB, feeding IEP increased total concentrate consumption (*p* = .001) by 3.6kg/calf. Over the entire study, daily water intake was higher (*p* = .002) for the IEP group when compared with the PCB group. In the IEP group, 12 calves were scored as scouring whereas there were 14 calves in the PCB group. There were no significant differences between treatments in faecal pathogen load of neither healthy nor scouring calves. In conclusion, supplementing IEP during the first 14 days of calf life improved the performance of newborn calves. Further work is warranted to understand the mode of action of IEP in calves.

## 1 INTRODUCTION

One of the major issues in newborn calves is diarrhoea. Neonatal diarrhoea can have several infectious causes such as *Cryptosporidium parvum*, *E. coli*, *Salmonella*, rotavirus and coronavirus, or combinations of two or more pathogens (Holland, 1990; Lorenz et al., 2011; McGuirk, 2008; Muktar et al., 2015). Newborn calves are more susceptible to such infections as they have a naïve immune system and are not yet able to react to specific antigens. In nature, this is overcome via passive immunity, in which immunoglobulins (Ig), mainly IgG, are transferred via the colostrum to the animals. In commercial practice, calves are provided colostrum during the first days of life. However, as neonatal diarrhoea occurs mainly during the first month of life, it could be considered that feeding immunoglobulin‐rich colostrum during the first days of life does not protect sufficiently against neonatal diarrhoea. The immunoglobulins in the colostrum do not provide protection for long enough considering the underdeveloped immune system of the neonatal calf. The gap between the colostrum provision and the full development of the immune system should be overcome.

The principle of passive immunity in young animals is not only used by cattle, but also for other species such as chickens (Rose et al., 1974). Chickens transfer immunoglobulins, mainly IgY, via the egg yolk where it can be used by the chick. In the 1970s, the presence of IgA and IgM was confirmed in the egg white (Rose et al., 1974; Yakamoto et al., 1975). The IgY in the egg yolk contains the avian equivalent of mammalian IgG (Chalghoumi et al., 2009). Immunization of the hen results in production of specific IgY which is transferred to the egg (Lösch et al., 1986). Specific IgY can be obtained against bacteria, viruses and also parasites (Gottstein & Hemmeler, 1985). Amongst others, specific antibodies against *Cryptosporidium parvum* can be produced in the egg. Feeding these egg antibodies resulted in a decreased *C. parvum* load in mice, providing evidence that egg antibodies have the potential to reduce infection load (Cama & Sterling, 1991).

In the current study, freeze‐dried whole egg, including yolk and egg whites, were fed to newborn calves during the first 14 days of life. The egg products originated from hens hyperimmunized against the most common pathogens causing neonatal diarrhoea in calves: *Cryptosporidium parvum*, *E. coli* (K88, K99, 987P and F41P), *Clostridium perfringens* (types A, C and D), Rotavirus, Coronavirus and *Campylobacter fetus‐jejuni*. The objective of this study was to evaluate the effect of these egg products on the health and growth performance of newborn dairy calves. It was anticipated that these egg products would help the calves to overcome neonatal diarrhoea resulting in healthier calves that would outperform calves that did not receive the egg products.

## 2 MATERIAL AND METHODS

### 2.1 Animals

The study was conducted at the Agri‐Food and Biosciences Institute (AFBI), Hillsborough, Northern Ireland (latitude 54°27’N; longitude 06°04’W). All experimental procedures in this study were conducted under experimental license granted by the Department of Health, Social Services & Public Safety for Northern Ireland (DHSSPSNI) in accordance with the Animals (Scientific Procedures) Act 1986. A total of 64 Holstein calves (32 males, 32 females), born between 12 January and 6 March 2017 with average birth weight of 40 kg (± 4.5 kg), were sourced from the AFBI dairy herd. All calves remained with their dam for a maximum of 13 hr after birth before being moved to straw bedded individual housing with an average air temperature of 13.5ºC (−0.4ºC to + 24.7ºC) and relative humidity of 67% rH (31.1 to 98.1%rH).

### 2.2 Diet treatments

All animals received colostrum for the first 3 days of life after which colostrum was replaced with calf milk replacer (CMR). The CMR provided was a commercial product (Milkivit Premium Plus, without Pulmo+, Trouw Nutrition) with the composition as presented in Table 1. The CMR (150 g/L) was provided at a rate of 15% of birth weight (BW) until day 49, which was reduced to 10% of BW between days 49 and 57, and 5% between days 57 and 63 and the calves were weaned at day 63. The calves remained on study until 70 days of age. Along with the CMR, calves had ad libitum access to concentrates (Table 2), chopped straw and fresh drinking water throughout the experimental period. The first 23 calves enrolled in the study were allocated 15% BW milk allowance and were not enrolled on a buildup strategy of the milk replacer; however, all remaining calves were assigned to a milk replacer buildup scheme from 10% BW increasing to 15% BW by 14 days of age.

**TABLE 1:** Composition of the calf milk replacer on dry matter basis used during the study

Nutrient	Content
Total Milk Solids, %	70
Fat (measured as ether extract), %	17
Crude protein, %	23
Crude fibre, %	0.05
Ash, %	8
Gross Energy content, MJ/kg	20.3
Vitamin A, iu/kg	50,000
Vitamin D3, iu/kg	6,000
Vitamin E, mg/kg	500
Selenium, mg/kg	0.36
Copper, mg/kg	13

**TABLE 2:** Composition of the concentrates on fresh basis used during the study

Nutrient	Content (%)
Maize	21
Barley	20
Soya	19
Wheat	11
Sugar beet	10
Soya hulls	7.5
Rapeseed	5
Molasses	3
Flour	1.2
Salt	0.8
Palm oil	0.75
Dairy mineral	0.4
Gross energy (MJ/kg)	18.1

Calves were balanced across treatment groups for birth weight and sex. Treatments consisted of a gut health additive containing immunized egg proteins (IEP) originating from hens vaccinated against *Cryptosporidium parvum*, Rotavirus (group A and group C), Coronavirus, *E. coli* (K88, K99, 987P and F41P), *Clostridium perfringens* (type A, type C and type D) and *Campylobacter fetus‐jejuni* (Trycop, Trouw Nutrition, The Netherlands) or a placebo supplement (PCB) containing dextrose and whey powder to correct for energy and protein (prepared and provided by Trouw Nutrition, The Netherlands), both of which were administered for the first 14 days of life. The IEP group consisted of 17 females and 15 males, whereas in the PCB group, 15 females and 17 males were included. The calves received one feeding of colostrum containing 30 g of the treatment product. Thereafter, 10 g of treatment product was added to each colostrum or CMR feeding, resulting in 20 g per day over the two daily feedings. No additives were included within the CMR feed after day 14.

### 2.3 Performance measurements

Live weights were recorded weekly according to individual calf age between 7 and 70 days of age. Weighing occurred between 2 and 4 hr post morning milk feed and was conducted using a calibrated mobile weighbridge (Tru‐Test Eziweigh 5, Auckland, New Zealand). The live weights were used to calculate the average daily gain (ADG). Individual daily CMR and concentrate intake were recorded on a daily basis, and drinking water intake was recorded 5 days/week throughout the experimental period. The general health of all calves was monitored daily, and any issues identified were treated according to standard operating procedures.

### 2.4 Faecal scoring and sampling

During the first 28 days of life, faecal consistency was visually assessed on a daily basis using a 4 point scoring system whereby 1 = normal consistency, 2 = slightly liquid consistency, 3 = moderately liquid consistency and 4 = primarily liquid consistency (Quigley et al., 2006). A calf was recorded as scouring at a score of 3 or 4. Faecal scoring was carried out by a trained technician. Scouring calves were treated by supplementing electrolytes (Diakur, Boehringer Ingelheim International GmbH, Ingelheim am Rhein, Germany) into the milk feed for 3 days. Calves that showed more severe scouring were treated with electrolytes in the water, as two additional daily feeds and a 3‐day course of antibiotics. Rectal faecal samples were collected weekly, on days 1, 7, 14, 21 and 28, for pathogen determination. Where scouring occurred within the first 28 days of life, additional faecal samples were collected during the first 4 d of scouring. Faecal samples were stored at 4°C prior to analysis. All faecal material collected was split into two separate aliquots. A minimum of 10g of fresh faeces was oven dried at 85ºC for 48 hr for determination of dry matter content. The second aliquot of faeces was submitted to the laboratory for analysis of pathogen levels.

### 2.5 Pathogen analysis

Pathogen analysis of the faeces was conducted weekly and included up to 20g faecal material tested for bacterial (*E.coli*, *Salmonella*), viral (Rotavirus, Coronavirus) and *Cryptosporidium* counts. Bacteriology analysis was carried out according to ISO 16649–2 for *Escheria coli* 0,157:H7 and ISO 16,579 for *Salmonella* detection. Virology analysis included the nucleic acid detection of Bovine Rotavirus and Coronavirus via Reverse transcription polymerase chain reaction (RT‐PCR) on the AB 7,500 Taqman system (Thermo Fisher Scientific). Presence of *Cryptosporidum parvum* in the faecal samples was analysed using immunofluorescent antibody test (IFAT) followed by microscopy assessment to count oocysts per gram (OPG).

### 2.6 Statistical analysis

All data were tested for normality and analysed using GenStat 16.2 (VSN International) for PC/Windows 7. All statistical models included birth weight (to account for initial variation), sex and CMR buildup strategy as covariates along with the fixed effects of time, CMR treatment and associated interactions unless otherwise stated. Housing block was included as a random term across all models. Time was included as repeated measure for the growth performance and feed intake results. Significance was determined via REML analysis with variance components incorporated using a linear mixed model. Following repeated measures analysis of calf performance measures, differences between treatments at specific time points were detected via pairwise *t* test. Probability of *p* < .05 was selected as the level of significance. Data were then subjected to Fisher's unprotected least significant difference (PLSD) test. Daily intakes, total intake, body weight, average daily weight gain, faecal score, faecal dry matter and pathogen load data were included in the analysis.

## 3 RESULTS

### 3.1 Performance

**FIGURE 1:**
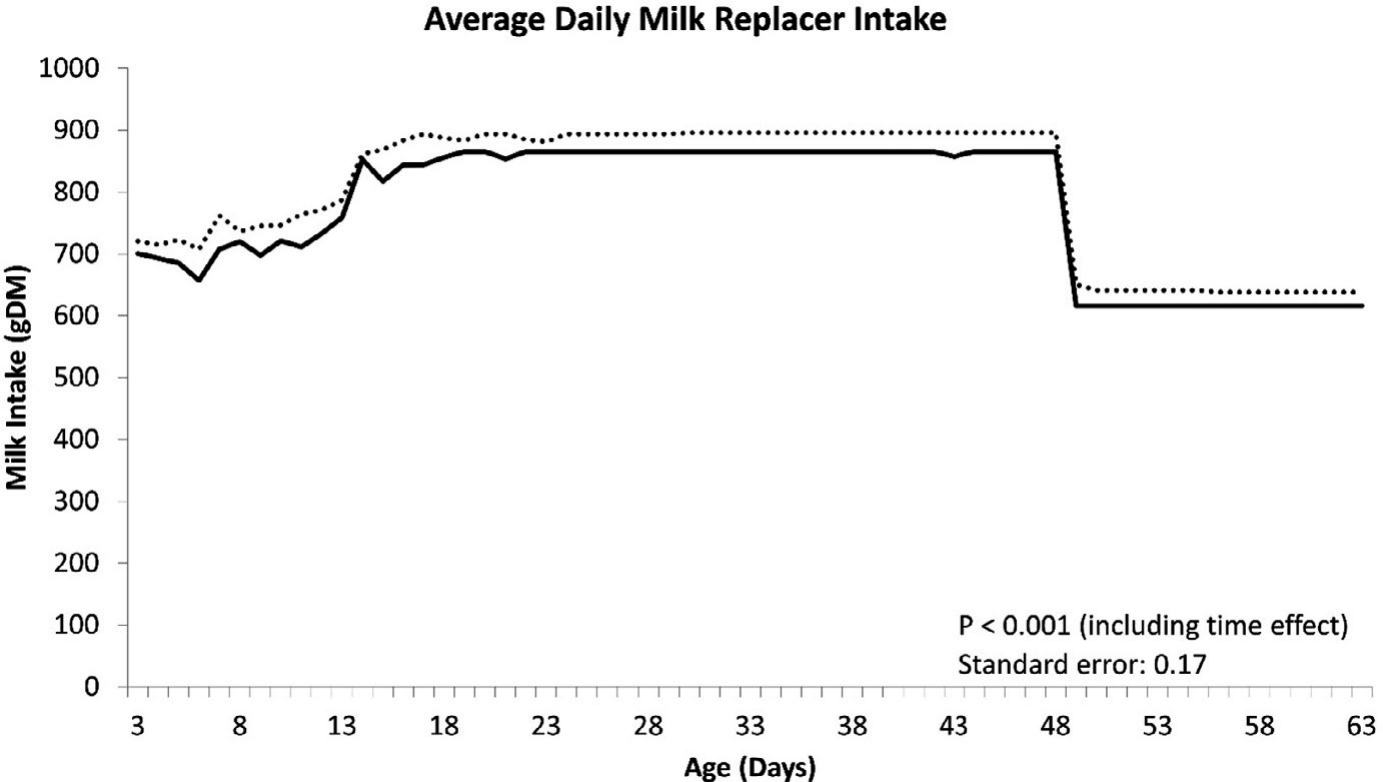
The average daily milk replacer intake per calf for each day until weaning. Dotted line represents the immunized egg protein (IEP) group, and the solid line represents the placebo (PCB) group

**FIGURE 2:**
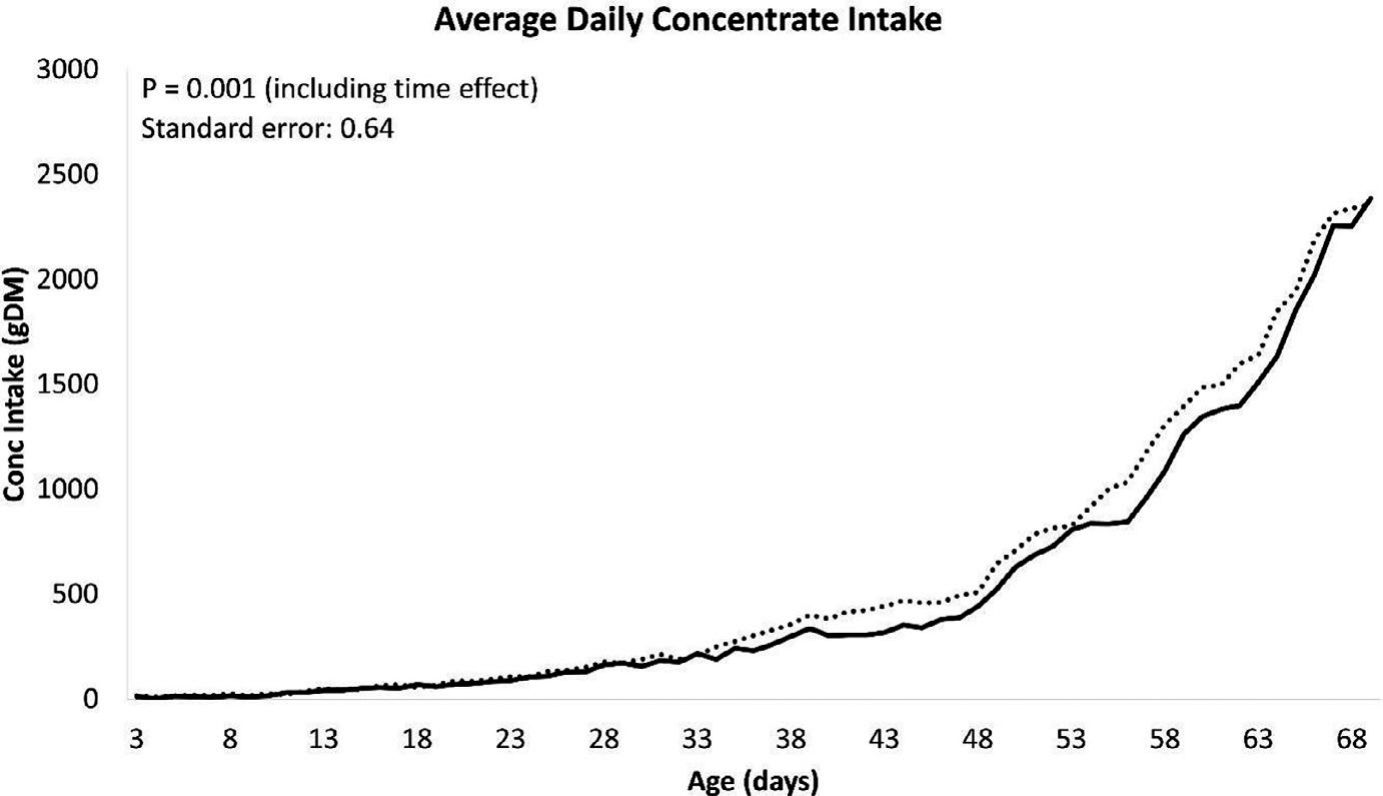
The average daily concentrate intake per calf per day during the entire study. Dotted line represents the immunized egg protein (IEP) group, and the solid line represents the placebo (PCB) group

**FIGURE 3:**
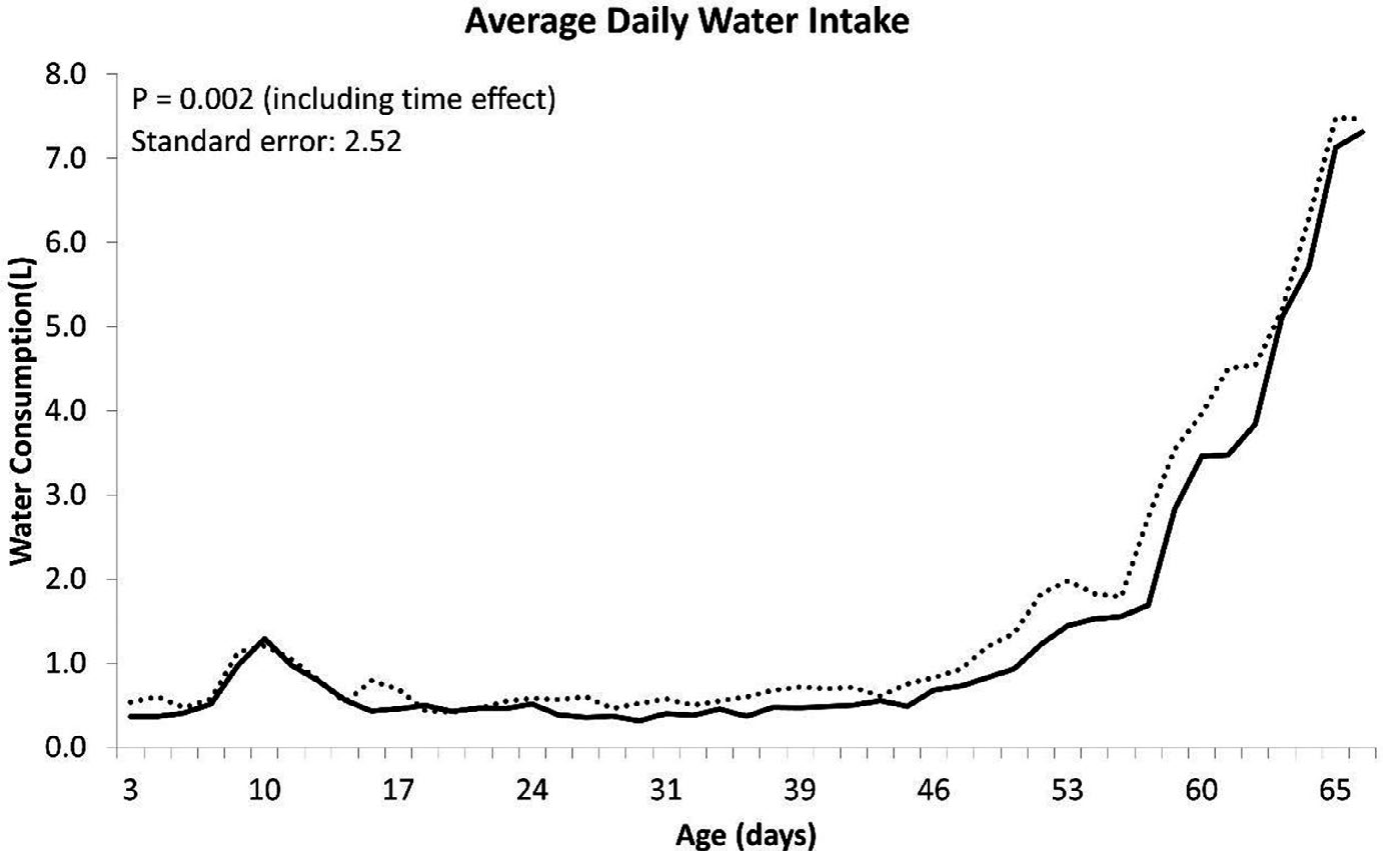
The average daily water intake per calf per day during the entire study. Dotted line represents the immunized egg protein (IEP) group, and the solid line represents the placebo (PCB) group

The growth performance of calves is presented in Table 3. Body weight was significantly higher at d 42 (*p* = .03) and d 56 (*p* = .02), and tended to be higher at d 28 (*p* = .06), d 49 (*p* = .05) and d 63 (*p* = .06) in IEP calves when compared with PCB calves. At the end of the study on d70, IEP calves were, on average, 4 kg heavier than the PCB calves (*p* = .06). There were no significant differences in ADG between treatment groups, with IEP and PCB calves achieving 0.67 kg/day and 0.63 kg/day, respectively, between birth and 70 days of age. Taking into account the slight, but non‐significant, difference in birth weight at the start of the study, repeated measures analysis indicated growth performance was not significantly (live weight d 70: *p* = .614, ADG d0 to d70: *p* = .485) different between the treatments. Average daily CMR, concentrate and water intake are presented in Figures 1, 2 and 3, respectively. Milk replacer was fed as a percentage of body weight; however, a milk buildup scheme was subsequently introduced to permit calves to consume the required volume. The first 23 calves enrolled in the study were allocated 15% BW milk allowance and were not enrolled on a buildup strategy of the milk replacer; however, all remaining calves were assigned to a milk replacer buildup scheme from 10% BW increasing to 15% BW by 14 days of age. There was no significant difference between treatments in total CMR intake (Table 4; *p* = .804), including a correction of the CMR buildup strategy. Calves supplemented with IEP consumed 3.6kg DM more than PCB calves between d3 and d70 (Table 4; *p* = .025). Average daily water consumption was significantly (*p* < .001) greater for IEP calves during the study (Figure 3), who consumed approximately 14.6L more than PCB calves during the entire study. However, the total amount of water consumed was variable; therefore, the overall treatment difference was not considered significant (*p* = .123) unless analysed with a repeated measures analysis (*p* = .002) (Table 4).

**TABLE 3:** The body weight and average daily gain per calf measured weekly throughout the experimental period

Age, Days	Body weight, kg				Average Daily Gain (ADG)^a^, kg/day			
	IEP	PCB	SE	*p*‐value	IEP	PCB	SE	*p*‐value
Birth	41.3	39.6	0.73	0.085				
7	44.2	42.8	0.80	0.22	0.41	0.46	0.06	0.51
14	44.8	42.9	0.83	0.13	0.25	0.24	0.04	0.90
21	48.3	46.2	0.95	0.09	0.33	0.31	0.03	0.64
28	52.1	49.5	0.94	0.06	0.38	0.36	0.02	0.38
35	56.7	54.8	1.00	0.14	0.44	0.43	0.02	0.81
42	62.2	59.1	1.09	0.03	0.50	0.47	0.02	0.15
49	66.9	64.0	1.14	0.05	0.52	0.50	0.02	0.25
56	73.6	69.5	1.34	0.02	0.58	0.53	0.02	0.08
63	80.2	76.5	1.42	0.06	0.62	0.59	0.02	0.24
70	87.9	83.9	1.48	0.06	0.67	0.63	0.02	0.23

Abbreviations: IEP, immunized egg protein treatment group; PCB, placebo treatment group; SE, standard error.

^a^ Average daily gain between birth and days of age.

**TABLE 4:** Cumulative feed intake throughout the experimental period (d 3–63)

	Treatment		SE	*p*‐value	
	IEP	PCB		Treatment	Treatment × Day
CMR (kgDM)	48.5	47.9	0.17	0.804	<0.001
Concentrate (kgDM)	34.5	30.9	0.64	0.025	0.001
CMR + concentrate (kgDM)	83.0	78.9	0.65	0.034	<0.001
Total drinking water (L)	80.3	65.7	2.52	0.123	0.002

Abbreviations: CMR, calf milk replacer; IEP, immunized egg protein treatment group; PCB, placebo treatment group; SE, standard error.

### 3.2 Faecal scores and faecal consistency

In total 12 calves in the IEP group and 14 calves in the PCB group were recorded as having experienced scour. All scouring occurred within the first 3 weeks of life. Additionally, the dry matter content of the faecal samples from the IEP group was 18.5%, whereas this was 17.8% in the PCB group (*p* = .502). However, due to the large variations among the calves (3.8%–52.4%), this difference was not significant.

### 3.3 Pathogenic load

Results from faecal samples submitted for microbiological analyses are presented in Table 5. Although not significant, in the IEP group numerically slightly more calves (27.5%) excreted *Cryptosporidium parvum* compared to the PCB group (22.5%). Virology results show that Rotavirus was observed in most calves and was excreted by numerically more calves in the IEP group (70.6%) compared to the PCB group (65.6%). Coronavirus was the only pathogen that was excreted by numerically less calves in the IEP group (5.0%) compared to the PCB group (6.9%). Bacteriological results show that there was no *Salmonella* present in any of the calves. *E. coli* was excreted by 56.9% of the calves in the IEP group and by 55.0% calves in the PCB group. None of the results regarding pathogenic load were significantly different. From all calves that were identified as experiencing scour, 12 in the IEP group and 14 in the PCB group, additional faecal samples were taken during the first 4 d of scouring. In the scouring calves, similar trends compared to the total population were observed. Also in the scouring group of calves, there were no significant differences observed in excretion of any pathogen.

**TABLE 5:** The number of calves in which the pathogen was detected above cut‐off level. The numbers in brackets are the scouring calves in which the pathogen was detected above cut‐off level

		Parasitology	Virology		Bacteriology	
		*C. parvum*	Rotavirus	Coronavirus	*Salmonella*	*E. coli*
Positive cut‐off level		>1,000 OPG	>0 Ct RNA	>0 Ct RNA	Qualitative	1 × 10^7^ CFU/g^a^
	Age, days					
Immunized egg protein (*n* = 32)	1	1 (1)	7 (3)	1 (1)	0	28
	7	9 (6)	27 (12)	1 (1)	0	21
	14	23 (10)	30 (11)	2 (1)	0	18
	21	3 (2)	26 (12)	1 (1)	0	8
	28	8 (2)	23 (9)	3 (2)	0	16
	Total %^b^	27.5	70.6	5.0	0	56.9
Placebo (*n* = 32)	1	5 (2)	5 (4)	0	0	27
	7	5 (3)	28 (13)	0	0	23
	14	17 (8)	24 (12)	7 (4)	0	17
	21	4 (0)	27 (12)	0	0	12
	28	5 (3)	21 (11)	4 (1)	0	9
	Total %^b^	22.5	65.6	6.9	0	55.0

^a^ Determined from mean level detected.

^b^ Total %=percentage positive of all samples taken.

## 4 DISCUSSION

The aim of this study was to examine the effect of immunized egg proteins on neonatal diarrhoea and performance of newborn calves. At the point of weaning, body weight was 4kg higher in calves supplemented with IEP in the first 14 days of life than those calves provided with PCB. As a result, the ADG seemed higher for the IEP group although not significant, which may be related to the differences in initial body weight. As calves were allocated to the study as they were born, there was variation of body weight between treatments; however, this was accounted for within the analysis of growth performance data by using birth weight as a covariate in the statistical model. The mechanisms behind the improved body weight in the IEP group remain unclear from the current study, because the hypothesized effect of IEP on pathogen and scouring could not be confirmed. This study showed no significant impact on the presence of the focus pathogens, such as *Cryptosporidium parvum*, Rotavirus, Coronavirus or *E. coli*, nor did the scouring incidence.

Previous work by Ikemori et al. (1992) showed a lower mortality in newborn calves that were fed egg antibodies against *E. coli* compared to calves fed the control diet, suggesting that there could be a positive impact of providing egg antibodies to neonatal calves. As reported by Diraviyam et al. (2014), eggs from hyperimmunized hens contain specific antibodies (IgY) against specific pathogens to which the hens were vaccinated, the use of which has been described for calves, poultry and swine. The meta‐analysis indicated a consistent positive effect of the IgY on diarrhoea and mortality by diarrhoea in poultry, swine, mice and calves. Although the individual studies included in the meta‐analysis did not always show significant benefits, an overall positive effect of IgY was observed, particularly with regard to its use in calves. The results of the current study are similar, as although diarrhoea occurrence was not significantly reduced in IEP calves, numerically fewer calves experienced diarrhoea.

In vitro results show that egg proteins were able to prevent adhesion of *E. coli* to the intestines by acting on the fimbriae (Yokoyama et al., 1992) and even cause inhibition of disease through direct binding of bacteria (Marcq, Thewis, Portebelle & Beckers, 2010). This suggests a direct mode of action of IgY in the intestines rather than a required uptake of the IgY into the bloodstream, which was confirmed by Quezada‐Tristán et al. (2014). However, in the present study immunoglobulin level in the blood was not determined. Additionally, the microbiology results could not confirm this, with no difference in pathogen/bacterial excretion observed. Similarly, IgY specifically against bovine rotavirus resulted in some growth benefits and a delated start and shorter duration of diarrhoea responses were observed in rotavirus challenges calves (Vega et al., 2011). However, the shedding of rotavirus was not decreased in the animals receiving dietary specific IgY leading the authors to conclude that IgY from vaccinated hens seemed to prime the neonatal mucosal immune response to bovine rotavirus. Although a less severe or delayed diarrhoea was not observed in the current study, the numerically lower frequency of diarrhoea occurrence in IEP calves could be suggestive of a subclinical effect of the egg proteins.

If the pathogens cannot adhere to the intestine, they will be excreted without causing problems meaning that excretion will be the same regardless of IgY supplementation (Xu et al., 2011). The difference would be in the viability of the pathogens. The type of microbiological measurements used in the current study for faecal pathogen load did not discriminate on microbial viability.

The pathogen load analysis showed that different pathogens were present under the current, non‐challenged, circumstances. This is in line with work by Holland (1990) who described that most infections have multiple causes. Also, the type of infections change in the course of life, where in the first 5 days of a calves life mostly *E. coli* can be expected, between 5 and 14 days of age infections with rotavirus, coronavirus, *Salmonella* and *Cryptosporidium parvum* can be expected (Lorenz et al., 2011; McGuirk, 2008). For these reasons, IgY from hyperimmunized eggs should target more than one pathogen to be effective. Based on the prevalence of pathogens described in literature, animals need at least protection during the first 14 d of their lives.

In addition, IgY is not the only compound described in eggs to have immune modulatory effects (Vega et al., 2012). Both the egg white and egg yolk contain various biologically active components as summarized by Mine and Kovacs‐Nolan (2004). As the IEP treatment contained whole freeze‐dried eggs including both the egg white and the egg yolks, more active components other than just IgY may have been present.

Scouring is often associated with malabsorption of nutrients from the diet (Holland, 1990). In the current study, the IEP group ate more concentrates and calf live weight was also increased to a similar extent. Possibly, the calves had less problems with malabsorption from the diet because IgY could improve gut integrity and function, thus have more nutrients better digested and absorbed, resulting in improved growth performance. The variation in body weight gain of calves can be explained for 49% by the starter intake (Kertz, Reutzel & Mahony, 1984). Another possible reason for the better performance could be related to a subclinical effect of IEP resulting in better health conditions of the animals. Providing egg proteins appears to prime the neonatal mucosal immune response (Vega et al., 2011) which may help the calf to deal with enteric diseases early in life, and in this way could have the potential to improve the growth for a longer period. No immune response markers were analysed in the current study to confirm any subclinical effects. Soberon et al. (2011) reported that each kilogram of additional ADG pre‐weaning may result in a higher milk yield of at least 850 kg in the first lactation. This may be the result of a long lasting effect of higher starter intake, as exposure of calves to concentrates before weaning results in a higher concentrate intake post weaning (Xiao et al., 2018). Future studies in which the calves are followed until first lactation should be considered to investigate the impact of pre‐wean feed intake.

Interestingly, the calves in the IEP group drank more water than the calves in the PCB group. Kertz et al. (1984) showed that water intake was a result of scouring rather than a cause. This higher water intake suggests that the calves in the IEP group could deal better with the scouring.

The results of the current study indicate that feeding immunized egg proteins to calves in the first 14 days of life improves growth performance and concentrate intake. Although no significant health benefits were observed, further research should be done on possible subclinical health effects of immunized egg proteins.

## ANIMAL WELFARE STATEMENT

The authors confirm that the ethical policies of the journal have been adhered to, according to the guidelines of the journal. All experimental procedures in this study were conducted under experimental license granted by the Department of Health, Social Services & Public Safety for Northern Ireland (DHSSPSNI) in accordance with the Animals (Scientific Procedures) Act 1986. The research meets the EU standards for the protection and use of animals for scientific purposes and/or feed legislation.

## Data Availability

Research data are not shared.
